# Bilious Fever with Torpor of Brain

**Published:** 1858-11

**Authors:** W. R. Stone, R. B. Dewey

**Affiliations:** Manhattan; Pleasant Garden, Ind.


ARTICLE III.

### BILIOUS FEVER WITH TORPOR OF BRAIN.

BY W. R. STONE, OF MANHATTAN, AND R. B. DEWEY, OF PLEASANT GARDEN, IND.

CASE 1.—J. H., aged 50 years, of bilious temperament, a farmer of industrious habits, was afflicted during the summer of 1855 with intermittent fever, which was, from time to time, relieved by sul. quinia, up to Sept. of the same year, when the disease assumed a different and somewhat singular character, he being disposed to sleep each day at the time his paroxysms of *fever* had formerly manifested themselves. Each day this disposition increased, but was lightly regarded by himself, against the warnings of his physician, W. R. Stone, for one week, when his wife called on the Dr. in the morning, stating that her husband could not be awakened, and requested the Dr. to visit, upon doing which, he was found as follows: pulse 80, small and compressible; skin cool and dry; respiration slow and rather laboring; head of equal temperature with the body. He was, at this time, in a profound slumber, out of which it was impossible to arouse him till the aid of a sinapism to the nuchial region was obtained, when he was sufficiently aroused to take medicine.

℞ Sul. quin., grs. 100, divided into twelve powders, one of which to be taken every two hours till he is more wakeful, when the doses may be halved, and time lengthened to three hours. Some 60 grs. were taken before this effect was induced, when small portions of proof spirit was alternated with the quinia.

The subsequent treatment, to perfect recovery, consisted in tonic doses of quinia and sesqui-carb. of iron, by which the patient was entirely restored in three weeks.

Removing to a distant part of the country, in about one year he was again attacked just as before, when a medical attendant was procured, who waged a merciless warfare against quinine as an agent impotent to good offices, but fruitful in evil deeds, when, as a matter of course, its use was dispensed with in the case. The patient slept near one week, and then died for want of quinine.

CASE 2.—T. K. S., aged 50 years, of nervous temperament, a farmer, and of industrious habits, complained for some days of pain, and tenderness in right hypochondrium, with uneasiness of the stomach, bowels constipated.

On the morning of June 20th, 1857, R. B. Dewey was called to see him, the report being, that on arising at day-light he became blind, dizzy, deaf, and lost his speech.

Repairing to his house he was found as follows: pulse 40, small and compressible; surface cold, and of an icteroid hue; pupils contracted; no preternatural heat of head; voice weak, and employed with much difficulty as though a heavy weight were pressing the chest. The lingual muscles were partially paralyzed, causing pronunciation to be imperfectly performed.

Viewing the case as one of miasmatic origin, in which the weight of the attack had fallen on the nervous system, which was deemed free of organic lesion, he was put upon the following:

| | | |
|---|---|---|
| ℞ | Sul. quin., | grs. 50. |
| | Pulv. capsic., | " 10. |

Mix, and divide into six powders, one to be taken every two hours. Six grs. of sub. mur. hydr. was given with first powder. Frictions, with stimulating pediluvia to the extremities, together with a sinapism to the nuchial region were employed. Under this treatment the patient soon regained his health.

CASE 3.—We visited this patient together on the 22d of May, 1858, and found him rather plethoric, of bilious temperament, a farmer, and 27 years of age. Condition: pulse 130, and moderately compressible; tongue slightly pale and flabby; skin somewhat yellow and moist; pupils dilated and intolerant of light; severe pain in the head; bowels constipated; nausea, and vomiting of bile. No preternatural heat of head.

*Previous History.*—Six months back the patient became afflicted with periodical neuralgia in the form of hemicrania, which had been temporarily relieved from time to time, till within one month of the present attack, when he was taken severely with what seemed to be neuralgia of head and face, together with great functional derangement of the stomach. He was promptly relieved with vegetable and mineral tonics, together with anodynes, and was requested to continue a tonic course for some time, which being neglected, the present attack supervened.

*Diagnosis.*—Remittent fever, with excitation of the nervous centres, of a debilitated character, in which we judged it imprudent to use depletives to much extent.

| | | |
|---|---|---|
| ℞ | Sub. mur. hydr., | grs. 12. |
| | Podophylin, | " 18. |

Mix, and divided into six powders, one to be taken once in three hours till bowels move, after which the following:

| | | |
|---|---|---|
| ℞ | Sul. quin., | grs. 35. |
| | Capsic. pulv., | " 8. |
| | Ferri. Prussias, | " 30. |

Mix, and divide into six powders, one of which every two hours.

Free catharsis was induced by second purgative powder, when, as anticipated, the pulse admitted of the use of the tonics, but the stomach still remaining irritable, it was rejected.

*May 23d.*—Feels better, from report of condition, but thinks another paroxysm of cephalalgia coming up. Sent him sul. morph. one grain, in four powders, one to be taken every four hours till quiet is produced.

*May 24th.*—Visited patient this morning, and found him in *all* respects much better. Left 40 grs. of quinine in acid solution, one-sixth to be taken every two hours. Also advised the steady use of quin. and iron for some weeks.

The patient is, at this date, May 30th, convalescing finely.

We had thought of offering some remarks upon this peculiar class of cases, but the length of our article admonishes us to close. We will, however, request the opinions of any of your readers in relation to those cases, which, so far as we know, are not mentioned in our practical works.

